# Determinants of non-adherence to diabetes treatment among patients at Hawassa University Comprehensive Referral Hospital, Ethiopia

**DOI:** 10.4314/ahs.v26i2.10

**Published:** 2026-06

**Authors:** Melisew Wuchew, Ashenafi Yirga, Dawit Ayele, Sileshi Melesse, Mohammed Omar Musa Mohammed

**Affiliations:** 1 Department of Statistics, College of Natural and Computational Sciences, Hawassa University, Ethiopia; 2 Discipline of Statistics, School of Agriculture and Science, University of KwaZulu Natal, Pietermaritzburg, Private Bag X01, Scottsville, 3209, South Africa; 3 College of Business Administration in Hawtat Bani Tamim, Prince Sattam Bin-Abdulaziz University, Al-Kharj, Saudi Arabia

**Keywords:** Adherence, Diabetes Mellitus, Antidiabetic Treatment, Multi-Method Tool, Ordinal Logistic Regression

## Abstract

**Background:**

Diabetes mellitus (DM) is a major metabolic disorder characterized by chronic hyperglycemia. Medication non-adherence remains a significant public health concern, particularly in sub-Saharan Africa, contributing to poor glycemic control and increased morbidity.

**Methods:**

This study assessed adherence using a multi-method tool, including self-reporting, a visual analog scale, pill identification tests, and pill counts. Data were collected from patients enrolled in the diabetes follow-up program at Hawassa University, Ethiopia, between 2008 and 2021. Ordinal logistic regression analysis was used to identify factors associated with treatment non-adherence using naturally ordered categorical data.

**Results:**

The multi-method tool revealed that patient adherence to antidiabetic drugs was 25.6% (low), 32.6% (moderate), and 41.8% (high). The analysis identified several significant predictors of adherence, including gender, depression frequency, knowledge about medication benefits, the quality of the relationship with care providers, the type of complications, satisfaction with care, educational level, and the frequency of participation in treatment decisions.

**Conclusions:**

Medication non-adherence remains a major challenge in diabetes management. Targeted interventions such as strengthening patient education, psychosocial support, and provider-patient communication, particularly for men and those with depressive symptoms, are essential to improve adherence and health outcomes.

## Background

Diabetes mellitus (DM) is a chronic metabolic disorder characterized by persistent hyperglycemia resulting from an absolute or relative deficiency of insulin, leading to disturbances in carbohydrate, protein, and fat metabolism^1^. As of the most recent estimates from the International Diabetes Federation (IDF) in 2021, approximately 537 million adults (20-79 years) worldwide are living with diabetes, which is expected to rise to 783 million by 2045 if current trends continue^2^,^3^. This represents a significant increase from earlier figures and highlights the growing diabetes epidemic globally. The prevalence of diabetes is now estimated at 10.5% of the adult population, underscoring a substantial public health challenge.

In addition, the IDF Diabetes Atlas^3^ notes that several factors, including urbanization, aging populations, increasing obesity rates, and lifestyle changes influence the rise in diabetes cases. These factors contribute to an increased risk of Type II diabetes and highlight the urgent need for comprehensive strategies to prevent and manage the disease effectively.

As of the latest data from the World Health Organization (WHO) and the International Diabetes Federation (IDF), diabetes was responsible for approximately 6.7 million deaths in 2021^3^. This translates to around 18,500 deaths each day, emphasizing the significant mortality burden associated with the disease.

Diabetes accounted for 12.2% of global deaths among adults aged 20 to 79 years in 2021. The increasing prevalence of diabetes, particularly Type II diabetes, coupled with associated complications, underscores the urgent need for effective prevention and management strategies. Several factors, including access to healthcare, management of co-morbid conditions, and socioeconomic determinants of health, can influence mortality rates. These figures illustrate the continued public health challenge posed by diabetes and the importance of ongoing efforts to reduce its impact on global mortality^3^.

According to the IDF Diabetes Atlas^3^, approximately 24 million adults aged 20-79 were living with diabetes in sub-Saharan Africa, which indicates a substantial rise since 2010. This region continues to experience one of the fastest-growing rates of diabetes globally. Projections suggest that by 2045, the number of adults with diabetes in sub-Saharan Africa could rise to around 47 million. The increase in diabetes prevalence has significant implications for mortality. In 2021, diabetes caused about 566,000 deaths in sub-Saharan Africa.

Furthermore, if current trends continue, this region is expected to face the highest increase in diabetes cases among all global regions, stressing the need for urgent public health interventions. These statistics underscore the increasing impact of diabetes not just as a health concern but also as a significant economic burden in terms of healthcare costs and loss of productivity, particularly in vulnerable regions like sub-Saharan Africa. Efforts to enhance diabetes prevention, management, and health systems are critical to address this growing challenge.

According to the International Diabetes Federation (IDF) Diabetes Atlas 10th edition (2021), Ethiopia had approximately 1.92 million adults aged 20-79 living with diabetes, representing a prevalence rate of 3.3%^3^. This figure indicates significant growth since 2000 and suggests that the country is experiencing a rising prevalence of diabetes. Projections estimate that the number of adults with diabetes in Ethiopia could reach around 3.5 million by 2045 if current trends continue. The increase in diabetes cases in Ethiopia is attributed to various factors, including lifestyle changes. Additionally, the country faces challenges related to healthcare access and diabetes management, which further complicate efforts to address this growing health concern. Furthermore, the burden of diabetes is not just limited to health outcomes; it also has economic implications, affecting productivity and healthcare costs. Addressing diabetes in Ethiopia will require comprehensive strategies focused on prevention, early detection, and effective management to mitigate the impact of this growing epidemic.

Identifying the determinants of non-adherence to diabetes management is crucial, as adherence is the extent to which a patient's behavior aligns with medical recommendations^4^. This process is characterized by the patient's active, voluntary, and collaborative involvement in a mutually acceptable course of behavior aimed at achieving therapeutic results. Adherence is not a static state but a dynamic and ongoing process requiring continuous support and awareness.

Although several studies have examined diabetes management in Africa, limited evidence exists on the determinants of non-adherence in Ethiopian settings. This study fills this gap by applying an ordinal logistic regression model to identify demographic, psychosocial, and clinical factors influencing adherence among diabetic patients at Hawassa University Comprehensive Referral Hospital in Ethiopia.

## Materials and Methods

### Data Collection and Study Design

This study focuses on patients enrolled in the diabetes follow-up program at Hawassa University Comprehensive Referral Hospital between 2008 and 2021. This hospital serves as a key healthcare facility in the region, providing specialized care for diabetes management. The antidiabetic treatment (ADT) registry database was utilized as the sole sampling frame for the research due to its comprehensive, accurate, and non-repetitive list of patients. This database includes detailed records of all diabetes patients aged 15 years and older who have been actively participating in the ADT medication program.

A total of 370 participants were selected for the study using a simple random sampling technique, as outlined by Cochran^5^. This method ensures that each patient has an equal chance of being included, enhancing the sample's representativeness. This systematic approach is critical for minimizing selection bias and ensuring the reliability of the findings.

In addition to demographic information, the data collected includes clinical parameters, adherence to treatment protocols, and associated health outcomes. This comprehensive dataset will provide valuable insights into the patterns of diabetes management in the study population and contribute to understanding the broader implications of diabetes care in Ethiopia.

### Study Variables

The primary outcome was adherence to antidiabetic treatment (ADT), categorized as low, moderate, or high using a multi-method assessment tool validated in similar resource-limited settings^6^. This tool combined a four-day self-report, pill count, visual analog scale, and pill identification test. This comprehensive approach allows for more accurate adherence measurement by capturing various aspects of patient behavior and medication management. Explanatory variables included: demographic Variables: (sex, and education), psychosocial Variables: (frequency of depression, and knowledge in the help of medication), clinical Variable: (complication types), and provider-related Variables: (frequency of participation in ADT decisions, satisfaction with care providers, and nature of the relationship with care providers).

Based on the above rating of the multiple data collection methods that include both qualitative and quantitative techniques (multi-method tool), the response variable is defined as:


(1) $${\text{Y}}_{\text{i}}=\left\{ \begin{array}{ll} 1, & \;\;\;\text{low adherence}\\ 2, & \;\;\;\;\;\text{moderate adherence}\\ 3, & \;\;\;\text{high adherence} \end{array}\right.$$

### Statistical Methods

The primary model utilized in this study is the ordinal logistic regression model, which is appropriate for outcomes with more than two ordered categories^7^,^8^. Among the different types of ordinal models, the proportional odds (PO) model is the most widely used^8^,^9^. This model estimates the probability of an event occurring and the likelihood of all events ranked before it on an ordinal scale. The model effectively captures the relationship between predictors and the ordered response variable by directly incorporating the natural ordering of response categories into the logits.

The cumulative probabilities represent the likelihood that the response variable falls within a specific category or any category below it, and c is the total number of categories. The cumulative probability of the **i**^**th**^ category is calculated as:


(2) $$Pr(Y\leq i)=p_{1}+p_{2}+\ldots +p_{i}=\sum_{k=1}^{i}p_{k}$$

This expression sums the probabilities of all categories up to, providing the cumulative probability for each possible outcome level.

The proportional odds (PO) model assumes that the cumulative logits of the response variable can be expressed as parallel linear functions of the independent variables^9^. This means that, under the PO assumption, the odds ratio is assumed to remain constant across all thresholds of the response variable.

The proportional odds (PO) model provides regression coefficients that are straightforward to interpret. The exponential of the coefficient, represents a uniform odds ratio across all cutoff points, effectively summarizing the influence of explanatory (*β*) variables on the response variable, in a single, commonly used measure. Because of its simplicity and clarity, the PO model is the most widely applied regression model for analyzing ordinal data^9^.

Let the cumulative probability of the first c-1 categories of **Y** is **pr(Y ≤ i) = π**_**i**_, for **i = 1,…,c − 1**. The odds for these cumulative probabilities can be expressed as:


(3) $$\text{odds}(Y\leq i)=\frac{pr(Y\leq i)}{1-pr(Y\leq i)}=\left[\frac{\pi_{i}}{1-\pi_{i}}\right];\;i=1,\ldots ,c-1$$

The log odds (logit) of these cumulative probabilities are given by:


(4) $$\text{log}\left[\frac{pr(Y\leq i)}{1-pr(Y\leq i)}\right]=log\left[\frac{\pi_{i}}{1-\pi_{i}}\right]$$

The relationship between the cumulative logits of **Y** is:


(5) $$log\left[\frac{\pi_{i}}{1-\pi_{i}}\right]=log\left[\frac{\pi_{i}}{\pi_{i+1}+\cdots +\pi_{c}}\right];\;i=1,2,\ldots ,c-1$$

Now, consider a set of **X** explanatory variables for subject **X**_**j**_^**′**^ **= (X**_**1j**_**, X**_**2j**_**, …, X**_**pj**_**)**, where ***j* = 1, 2, …, *n***. The relationship between the predictor and response variables is not linear in logistic regression. Instead, the PO model assumes a linear relationship between the logit of **pr(Y**_**j**_ **≤ i)** (i.e., the logit transformation of **π**_**i**_) and **X**_**j**_:


(6) $$\begin{array}{rl} log\left[\frac{pr(Y_{j}\leq i)}{1-pr(Y_{j}\leq i)}\right] & =log\left[\frac{\pi_{i}}{1-\pi_{i}}\right]=\alpha_{i}-[\beta_{1}X_{1j}+\beta_{2}X_{2j}+\ldots +\beta_{p}X_{pj}]\\ & =\alpha_{i}-X_{j}^{\prime }\beta \end{array}$$

where **β = (β**_**1**_**, β**_**2**_**, …, β**_**p**_**)**^**′**^ is a vector of unknown regression coefficients. An equation like this is solved for each category of the dependent variable, except the last one^9^.

The detailed explanation of the logistics regression model is well documented. This information can be accessed from books and articles in the literature^10^-^14^.

## Results

### Descriptive Statistics

Summary statistics of the various variables used in this study with respect to the current status of adherence to medication are given in Table 1 below:

**Table 1:** Descriptive statistics of adherence status of respondents cross-tabulated with associated covariates

Variables	Categories	Current Status of Adherence Level			Total	
		Low	Moderate	High		
		Count (%)	Count (%)	Count (%)		p-value
Sex	Male	58 (30.9)	64 (34.0)	66 (35.1)	188	0.031
	Female	35 (19.9)	60 (34.1)	81 (46.0)	176	
Frequency of depression	Always	17 (27.4)	30 (48.4)	15 (24.2)	62	0.002
	Frequently	38 (33.0)	35 (30.4)	42 (36.5)	115	
	Sometime	26 (20.5)	40 (31.5)	61 (48.0)	127	
	Never/little	12 (20.0)	19 (31.7)	29 (48.3)	60	
Educational level	No education	24 (50.0)	16 (33.3)	8 (16.7)	48	0.000
	Basic education	30 (35.3)	32 (37.6)	23 (27.1)	85	
	Elementary/second	28 (21.7)	47 (36.4)	54 (41.9)	129	
	Certificate and above	11 (10.8)	29 (28.4)	62 (60.8)	102	
Frequency of Participation in ADT decision	Never	30 (55.6)	17 (31.5)	7 (13.0)	54	0.000
	Sometime	30 (32.3)	36 (38.7)	27 (29.0)	93	
	Frequently	27 (19.1)	50 (35.5)	64 (45.4)	141	
	Always	6 (7.9)	21 (27.6)	49 (64.5)	76	
Knowledge in medication help	Strongly Agree	6 (9.4)	14 (21.9)	44 (68.8)	64	0.000
	Agree	28 (19.2)	54 (37.0)	64 (43.8)	146	
	Uncertain	34 (35.4)	33 (34.4)	29 (30.2)	96	
	Disagree	14 (33.3)	18 (42.9)	10 (23.8)	42	
	Strongly Disagree	11 (50.0)	5 (22.7)	6 (27.3)	22	
Nature of relationship with care-providers	Very bad	13(59.1)	4 (18.2)	5 (22.7)	22	0.000
	Bad	22 (37.3)	22 (37.3)	15 (25.4)	59	
	Average	13 (17.6)	35 (47.3)	26 (35.1)	74	
	Good	31 (23.3)	39 (29.3)	63 (47.4)	133	
	Very good	14 (17.3)	24 (29.6)	43 (53.1)	81	
Complication types	Kidney disease	12 (20.7)	19 (32.8)	27 (46.6)	58	0.016
	Nerve problems	13 (20.3)	25 (39.1)	26 (40.6)	64	
	Blood pressure	17 (25.8)	28 (42.4)	21 (31.8)	66	
	Eye disease	12 (21.8)	21 (38.2)	22 (40.0)	55	
	Heart failure	29 (43.3)	16 (23.9)	22 (32.8)	67	
	Others	10 (25.0)	11 (27.5)	19 (47.8)	40	
	No complications	4 (28.6)	5 (35.7)	5 (35.7)	14	
Satisfaction with care providers	Very unsatisfied	14 (58.3)	8 (33.3)	2 (8.3)	24	0.000
	Unsatisfied	19 (45.2)	14 (33.3)	9 (21.4)	42	
	Fairly satisfied	21 (41.2)	21 (41.2)	9 (17.6)	51	
	Satisfied	29 (23.0)	51 (40.5)	46 (36.5)	126	
	Very satisfied	10 (8.3)	30 (24.8)	81 (66.9)	121	

Note:* Total count of responses across the covariates might not match with the grand total count due to missing values.

Total missing values are < 5%. * *p-values were obtained using Pearson Chi-square test to assess significant difference between the response variable and nominal covariates (sex, and complication types) at the 0.05 significance level.* * *p-values were obtained using a Spearman's rho test to evaluate significant association between the response variable*

and ordinal covariates (frequency of depression, education level, frequency of participation in ADT decision, knowledge in medication help, nature of relationship with care-providers, and satisfaction with care providers) at the 0.05 significance level

From Table 1, we can see that 35.1% of males and 46.0% of females have a higher adherence status. On the other hand, 34% of males and 34.1% of females have a moderate status, and 30.9% of males and 19.9% of females have a low adherence status.

Table 1 presents the distribution of depression frequency among respondents, indicating that 24.2%, 48.4%, and 27.4% of patients reporting persistent depression fall into the higher, moderate, and low categories, respectively. On the other hand, 48.3%, 31.7%, and 20.0% of patients who reported having little or no depression are in the higher, moderate, and low statuses, respectively.

Results on “complication types” of respondents show that 46.6%, 32.8%, and 20.7% of those with kidney disease; 40.6%, 39.1%, and 20.3% of those with nerve disease; 31.8%, 42.4%, and 25.8% of those with blood pressure; 40.0%, 38.2% and 21.8% of those with eye disease; and 32.8%, 23.9% and 43.3% of those with heart failure are in the higher, moderate and low statuses, respectively. On the other hand, 71.4%, 28.6%, and 0.0% of those with no complications are in the higher, moderate, and low statuses, respectively.

Table 1 presents the results on respondents' satisfaction with care providers. Among those who reported being very unsatisfied, 8.3%, 33.3%, and 58.3% fell into the high, moderate, and low adherence categories, respectively. In contrast, among those who reported being very satisfied, 66.9%, 24.8%, and 8.3% were classified as high, moderate, and low adherence, respectively.

Results on “educational level” of respondents show that 16.7%, 33.3%, and 50.0% of those non-educated groups; 27.1%, 37.6%, and 35.3% of those having basic education; and 41.9%, 36.4% and 21.7% of those having elementary or secondary education are in the higher, moderate and low statuses, whereas 60.8%, 28.4% and 10.8% of those having certificate and higher education are in the higher, moderate and low statuses of adherence, respectively.

Treatment adherence also varies according to patients' level of in treatment decisions. As shown in Table 1, among patients with little or no involvement in ADT decision-making, 13.0%, 31.5%, and 55.6% demonstrated high, moderate, and low adherence, respectively. In contrast, among patients who consistently participated in ADT decision-making, 64.5%, 27.6%, and 7.9% were classified as high, moderate, and low adherence, respectively.

Table 1 also presents the Pearson Chi-square p-values for nominal categorical variables and Spearman's rho p-values for ordinal categorical variables, indicating the strength of association between predictors and adherence levels. All predictor variables examined in the study demonstrated significant associations with adherence levels (p-value < 0.05).

### Ordinal Logistic Regression Analysis

To explore the relationships between adherence levels and selected explanatory variables identified as statistically significant in the significant association analysis, we fitted an ordinal logistic regression (OLR) model. The statistical analysis was conducted using SAS 9.4 software.

### Assessing the Overall Goodness-of-Fit

The analysis shows that the selected explanatory variables significantly improve the model's ability to predict patient adherence levels compared to the intercept-only model, as indicated by the global null hypothesis test results. The highly significant p-values (p-value < 0.0001) for the likelihood ratio, score, and Wald tests indicate that the model as a whole fits significantly better than an intercept-only model, confirming that the inclusion of explanatory variables provides a meaningful contribution to predicting adherence levels (Table 2).

**Table 2:** Test results of the Global Null Hypothesis of the ordinal logistic model (BETA=0)

Test		Chi-Square	DF	p-value
Likelihood (LR)	Ratio	225.4869	28	<0.0001
Score		170.1136	28	<0.0001
Wald		139.4799	28	<0.0001

Moreover, the goodness-of-fit tests (Deviance and Pearson) indicate that the model fits the data well. Since the p-values are high and non-significant, we fail to reject the null hypothesis that the model fits adequately. The deviance and Pearson test results show non-significant p-values (0.9998 and 0.5357, respectively), suggesting that the model's fit to the data is acceptable. Thus, we conclude that the model reasonably approximates the observed data (Table 3).

**Table 3:** Deviance and Pearson Goodness-Of-Fit Statistics

Criterion	Value	DF	p-value
Deviance	548.0977	672	0.9998
Pearson	668.0581	672	0.5357

### Assessment of Linearity of Covariates

The score test results for proportional odds demonstrate that the null hypothesis of parallel logit surfaces is not rejected. With a p-value of 0.7676 (Chi-Square = 22.2914), the Score test indicates that the logit surfaces are parallel across adherence levels. This suggests that the proportional odds assumption holds, meaning that the effect of the covariates is consistent across the cumulative logits.

Based on the tests, strong evidence supports the proportional odds assumption in the model. Therefore, the ordinal logistic regression model is appropriate for analyzing adherence levels in this study, and the odds ratios across adherence categories are homogeneous.

The ordinal logistic regression analysis in Table 57 identified several significant determinants of non-adherence to diabetes treatment. Notably, male patients were more likely to exhibit non-adherence compared to female patients (OR = 1.856, p-value = 0.0035). Depression significantly impacted adherence; patients reporting frequent or consistent depression had higher odds of non-adherence, with odds ratios of 2.692 (p-value = 0.0257) and 2.865 (p-value = 0.0047), respectively.

**Table 5:** Parameter estimates of the ordinal logistic model

	Wald					95% CI for ()	
Parameter	Estimate	SE	Chi-Square	p-value			
Intercept 1	0.2068	0.0960	36.3663	<0.0001	-	--	
Intercept 2	0.0349	0.0899	14.0688	<0.0001	-	--	

Sex: [Ref.: Female]							

Male	0.6185	0.1105	6.1017	0.0035	1.856	1.494	2.305

Frequency of depression: [Ref.: Never or little]							

Always	0.9904	0.1583	4.9736	0.0257	2.692	1.974	3.671
Frequently	1.0525	0.0977	8.8654	0.0047	2.865	2.366	3.470
Sometime	0.3054	0.0616	0.7131	0.3984	1.357	1.203	1.531

Knowledge of medication help: [Ref.: Strongly agree]							

Strongly disagree	1.4063	0.0872	7.5517	0.0001	4.081	3.440	4.842
Disagree	0.6014	0.0764	18.6908	0.0107	1.825	1.571	2.120
Uncertain	0.7808	0.0461	35.2152	<0.0001	2.183	1.994	2.389
Agree	0.2941	0.0225	1.2553	0.1131	1.342	1.284	1.402

Nature of relationship with care providers: [Ref.: Very good]							

Very bad	1.1384	0.1026	8.5273	0.0035	3.122	2.553	3.817
Bad	0.2555	0.0994	1.5971	0.2063	1.291	1.063	1.569
Average	0.1406	0.1015	0.5857	0.4441	0.869	0.712	1.060
Good	0.1545	0.0913	0.8855	0.3467	0.857	0.716	1.025

Complication types: [Ref.: =No complication type]							

Kidney disease	0.8245	0.1003	4.6790	0.0305	2.281	1.889	2.754
Nerve disease	0.8046	0.0872	4.5263	0.0334	2.236	1.885	2.653
Blood pressure	0.6538	0.0521	3.1470	0.0076	1.923	1.736	2.130
Eye disease	0.8636	0.0784	5.3740	0.0204	2.372	2.034	2.766
Heart failure	1.3271	0.0831	12.5305	0.0004	3.770	3.203	4.437
Others	0.9576	0.1011	5.8742	0.0154	0.384	0.315	0.468

Satisfaction in care providers [Ref.: Very satisfied]							

Very unsatisfied	0.9966	0.1042	13.4624	0.0002	2.709	2.209	3.323
Unsatisfied	0.7070	0.1129	11.5048	0.0007	2.028	1.625	2.530
Fairly satisfied	0.6772	0.1101	12.1773	0.0005	1.968	1.586	2.442
Satisfied	0.5641	0.1092	15.2183	<0.0001	1.758	1.419	2.178

Educational level [Ref.: Certificate & above]							

No education	0.7881	0.1191	14.1444	0.0002	2.199	1.741	2.777
Basic education	0.5323	0.1436	9.5997	0.0019	1.703	1.285	2.257
Elementary/Second	0.2764	0.1028	3.3337	0.0679	1.318	1.078	1.613

Frequency of participation in ADT decision [Ref.: Always]							

Never or little	2.2702	0.0815	26.3321	<0.0001	9.681	8.252	11.358
Sometime	1.2316	0.0781	11.1973	0.0008	3.427	2.941	3.994
Frequently	0.7304	0.0663	4.5979	0.0320	2.076	1.823	2.364

Beliefs about medication efficacy were critical; patients strongly disagreeing with the effectiveness of their medication were 4.081 times more likely to be non-adherent (p-value < 0.0001). Similarly, patients with doubts about their medication's benefits showed significant non-adherence rates.

The patient-provider relationship was also influential. Those reporting very poor relationships with care providers were 3.122 times more likely to be non-adherent (p-value = 0.0035). Dissatisfaction with care further increased non-adherence risk; very dissatisfied patients had an OR of 2.709 (p-value = 0.0002).

Comorbidities played a substantial role. Patients with kidney disease (OR = 2.281, p-value = 0.0305), blood pressure-related complications (OR = 1.923, p-value = 0.0076), eye disease (OR = 2.372, p-value = 0.0204), or heart failure (OR = 3.770, p-value = 0.0004) were significantly more likely to be non-adherent.

Education level and participation in treatment decisions strongly influenced adherence. Patients with no formal education (OR = 2.199, p-value = 0.0002) or basic education (OR = 1.703, p-value = 0.0019) were less adherent than those with higher education. Additionally, patients excluded from treatment decisions faced nearly ten times higher odds of non-adherence (OR = 9.681, p-value < 0.0001).

These findings emphasize the importance of addressing educational disparities, enhancing patient-provider relationships, improving care satisfaction, and involving patients in decision-making. A multifaceted approach targeting these areas could significantly improve treatment adherence and patient outcomes in diabetes care.

## Discussion

The study highlights several key factors influencing patient adherence to diabetes treatment, including sex, diabetes-related complications, patient-provider relationships, satisfaction with care, and education levels. Studies suggest that men may have a greater tendency toward non-adherence. A study found that being male was linked to a lower chance of achieving remission and recovery, implying a higher risk of non-adherence in men^15^. Patients with complications, such as kidney disease, heart failure, or blood pressure issues, also showed lower adherence, aligning with findings by Bronas et al.^16^ that complex regimens and the progression of kidney disease reduce adherence due to fatigue and cognitive impairments.

Gender differences in adherence have been well-documented, with women generally more compliant than men^17^,^18^. This is likely due to higher health literacy and more proactive health behaviors among women. The impact of polypharmacy on adherence, especially among diabetic patients with hypertension, is also evident^19^, with the asymptomatic nature of hypertension reducing perceived urgency for medication^20^. Conditions like vision impairment, as noted by Fan et al.^21^, and the complexity of treatment regimens for heart failure^22^ are additional barriers to adherence.

The quality of patient-provider relationships plays a crucial role in treatment adherence. Patients with strong relationships with their care providers are more likely to adhere to treatment regimens, highlighting the importance of trust and effective communication^23^, ^24^. Conversely, poor communication or mistrust can lead to disengagement and reduced adherence^25^. Shared decision-making, as emphasized by Elwyn et al.^26^, empowers patients, aligns treatment with their values, and improves adherence.

Dissatisfaction with care provision is another critical factor influencing adherence. Patients who feel their care is inadequate are more likely to disengage from treatment^27^. This underscores the need for personalized care and improved communication to foster trust and enhance adherence^28^.

Education level also significantly impacts adherence. Uneducated patients are more likely to be non-adherent, highlighting the importance of health literacy interventions^29^,^30^. Tailored educational programs that simplify treatment instructions can improve adherence among those with lower literacy levels^31^.

The Necessity-Concerns Framework^32^ supports the finding that disagreement regarding medication effectiveness is a major predictor of non-adherence. Osterberg and Blaschke^33^ also emphasize that poor communication and adverse side effects can reduce patients' confidence in their treatment, leading to non-adherence.

Additionally, depression significantly impacts adherence. Studies show that patients with co-morbid depression are less likely to follow treatment protocols, exacerbating diabetes-related complications^34^,^35^. The interplay between diabetes and depression complicates management, making integrated care essential^36^.

Shared decision-making, as demonstrated by Stacey et al.^37^ and Thomas et al.^38^, enhances patient engagement and improves adherence by aligning treatment with patient values. Engaged patients are more likely to follow prescribed regimens, reinforcing the importance of participatory approaches^39^.

Overall, this study underscores the multifaceted nature of adherence, with factors such as patient-provider relationships, education, satisfaction, and depression playing significant roles. Future research should explore how these factors interact and inform comprehensive, patient-centered interventions to improve adherence and health outcomes.

The regression analysis confirms that sex, depression frequency, medication knowledge, patient-provider relationships, diabetes-related complications, care satisfaction, and education level are all significant predictors of adherence. Addressing these factors through improved communication, education, and targeted interventions could substantially enhance treatment adherence.

## Limitation of the Study

This study has limitations that should be acknowledged. First, its cross-sectional design restricts causal inference, as associations between explanatory variables and adherence cannot establish temporal or cause-and-effect relationships. Second, dependence on self-reported measures may introduce recall and social desirability biases, potentially affecting the accuracy of adherence estimates. Third, the single institution setting at Hawassa University Comprehensive Referral Hospital may limit generalizability to other Ethiopian or sub-Saharan African contexts. Finally, while ordinal logistic regression provided valuable insights, unmeasured confounders such as socioeconomic status or cultural factors may have influenced adherence behaviors. Future research employing longitudinal designs, multi-site samples, and broader contextual variables would strengthen understanding and inform more comprehensive interventions.

## Conclusions

This study identified key determinants of non-adherence to antidiabetic treatment among patients at Hawassa University Comprehensive Referral Hospital in Ethiopia, including gender, depression, education level, patient-provider relationships, and participation in treatment decisions. Based on these findings, recommendations are proposed to improve adherence. Healthcare providers should prioritize building supportive relationships with patients through effective communication, empathy, and trust. Patients with complications require targeted interventions, such as closer monitoring and specialized care, to ensure proper adherence. Enhancing patient satisfaction through feedback mechanisms and personalized care plans is also essential.

In addition, increasing awareness and promoting educational opportunities can strengthen health literacy and improve adherence, particularly among patients with lower education levels. Addressing these areas through focused interventions will contribute to better health outcomes. Beyond the clinical level, the findings highlight the need for broader public health and policy initiatives in Ethiopia as well as in sub-Saharan Africa, including strategies to reduce socioeconomic and cultural barriers to adherence and to expand access to quality care. Future research should employ larger, multi-site samples and longitudinal designs to explore additional determinants and evaluate the long-term impact of patient-centred interventions on adherence and health outcomes.

## Figures and Tables

**Table 4:** Score test for the proportional odds assumption

Chi-Square	DF	p-value
22.2914	28	0.7676

## Data Availability

The data used in this study are available upon reasonable request from Hawassa University Comprehensive Referral Hospital, Ethiopia. For inquiries, please contact Melisew W. Wuchew at melisewwww@gmail.com or meliseww@hu.edu.et.
